# Clinical significance of tumor location in non-muscle-invasive bladder cancer: A single-center longitudinal cohort analysis

**DOI:** 10.14440/bladder.2024.0053

**Published:** 2025-10-06

**Authors:** Caipeng Qin, Yun Peng, Fei Wang, Yuxuan Song, Yiqing Du, Tao Xu

**Affiliations:** 1 Department of Urology, Peking University People’s Hospital, Beijing 100044, China; 2 Peking University Applied Lithotripsy Institute, Beijing 100044, China; 3 Department of Urology, Beijing Jishuitan Hospital, Beijing 100035, China

**Keywords:** Non-muscle-invasive bladder cancer, Outcome, Recurrence, Tumor location

## Abstract

**Background:**

Recurrence of non-muscle-invasive bladder cancer (NMIBC) still presents a significant clinical challenge, with the contributing factors yet to be fully understood.

**Objective:**

This study explored the clinical implications of tumor location in NMIBC recurrence.

**Methods:**

An observational cohort study was conducted, including 108 NMIBC patients who experienced a total of 344 NMIBC diagnoses (both primary and recurrent) between 1999 and 2019. Clinical information was collected for primary and recurrent tumors. Tumor locations were classified into 10 categories: bladder neck (neck), dome, posterior, anterior, trigone, right, right rear, left, multiple site(s), and others. The association between tumor location and recurrence was systematically analyzed.

**Results:**

The median follow-up period lasted for 28 months (range: 2–88 months), the median recurrence interval was 13.5 months, and 44 patients (40.7%) progressed into muscle-invasive disease. Univariate analysis revealed that tumor location within the bladder significantly impacted recurrence-free interval, progression-free survival, and overall survival. Tumors situated in the bladder neck, dome, right posterior wall, and trigone demonstrated significantly shorter recurrence-free intervals, rendering these areas high-risk regions. The original tumor site was the most common relapse location, and the recurrence interval shortened as the number of recurrences increased. Over time, the recurrence pattern shifted, with tumors most frequently recurring in the left wall, multiple sites, right wall, and posterior wall.

**Conclusion:**

The findings suggest that bladder cancer most commonly recurs at the original site, with high-risk locations linked to shorter recurrent intervals and greater risks for disease progression. In addition, the recurrence interval tends to decrease with successive recurrences.

## 1. Introduction

Non-muscle-invasive bladder cancer (NMIBC), a urothelial condition, is characterized by the recurrent development of non-muscle-invasive tumors, some of which may eventually progress to invade the bladder wall musculature. The tendency for multiple recurrences is a hallmark feature of this cancer type.^1^ In this study, longitudinal analyses were conducted on patients with multiple recurrences, presenting a comprehensive profile of clinical features. A series of small cohort studies have postulated that tumor location correlates with patient outcomes, with sites such as the prostatic urethra, bladder neck, posterior wall, and trigone significantly being linked to shorter recurrence-free intervals.^2^,^3^ Other studies have shown that trigonal tumors were linked to higher risks of adverse pathology and that trigonal and bladder neck tumors were associated with increased odds of lymphatic metastasis and dome with pT3–4.^4^,^5^ Using data from a cohort of 108 patients who experienced a total of 344 NMIBC diagnoses, this study examined the relationship between tumor location and patient outcomes across multiple recurrences.

## 2. Materials and methods

This is a retrospective case study, and the requirement for ethical approval was waived by the hospital.

### 2.1. Patients

Data from 177 patients diagnosed between 1999 and 2019 were sourced from Peking University People’s Hospital. Sixty-nine patients (39%) with incomplete information, primary carcinoma *in situ*, or without a recurrence history were excluded from the analysis. The final analysis included a total of 108 patients, all of whom had undergone post-operative intravesical chemotherapy instillation (Table 1). Histopathological slides of all cases were reviewed by a referee pathologist to confirm tumor stage and grade. Each case had experienced at least one recurrence following primary tumor resection. Pre-operative cystoscopy provided detailed information on tumor location, and tumor locations were classified into 10 categories: bladder neck (neck), dome, posterior, anterior, trigone, right, right rear, left, multiple site(s), and others. All patients were followed up for a median of 28 months (range: 2–88 months). Disease progression was defined as undergoing radical cystectomy due to disease progression and/or death.

**Table 1:** Summary of the cohort

Parameters	Overall	Anterior	Dome	Left	Multiple	Neck	Posterior	Right	Right rear	Trigone
*n*	108	4	4	27	24	2	19	22	2	4
Age (year)	68.05 (11.75)	71.00 (11.63)	64.75 (17.37)	64.56 (11.28)	68.17 (12.96)	75.50 (12.02)	67.37 (11.39)	71.41 (10.59)	62.00 (16.97)	75.25 (6.40)
Gender										
Female	25 (23.1)	1 (25.0)	1 (25.0)	5 (18.5)	6 (25.0)	0 (0.0)	4 (21.1)	5 (22.7)	2 (100.0)	1 (25.0)
Male	83 (76.9)	3 (75.0)	3 (75.0)	22 (81.5)	18 (75.0)	2 (100.0)	15 (78.9)	17 (77.3)	0 (0.0)	3 (75.0)
Grade										
HG	66 (61.1)	3 (75.0)	3 (75.0)	14 (51.9)	15 (62.5)	2 (100.0)	15 (78.9)	10 (45.5)	1 (50.0)	3 (75.0)
LG	37 (34.3)	1 (25.0)	1 (25.0)	9 (33.3)	9 (37.5)	0 (0.0)	4 (21.1)	12 (54.5)	1 (50.0)	0 (0.0)
PUNLMP	5 (4.6)	0 (0.0)	0 (0.0)	4 (14.8)	0 (0.0)	0 (0.0)	0 (0.0)	0 (0.0)	0 (0.0)	1 (25.0)
T stage										
T1	24 (22.2)	1 (25.0)	2 (50.0)	6 (22.2)	8 (33.3)	0 (0.0)	3 (15.8)	3 (13.6)	0 (0.0)	1 (25.0)
Ta	84 (77.8)	3 (75.0)	2 (50.0)	21 (77.8)	16 (66.7)	2 (100.0)	16 (84.2)	19 (86.4)	2 (100.0)	3 (75.0)

Note: Age data are presented in mean (standard deviation [SD]), while gender, grade, and T stage data are listed in number (percentage).

Abbreviations: HG: High-grade; LG: Low-grade; PUNLMP: Papillary Urothelial Neoplasm of Low Malignant Potential; Ta: Non-invasive papillary urothelial carcinoma.

### 2.2. Statistical analysis

Kaplan–Meier analysis was performed using SPSS (version 27.0; IBM Corp., Armonk, NY, USA) to estimate progression-free survival and overall survival. The significance level was set at p<0.05.

## 3. Results

### 3.1. Primary and recurrent predilection sites of NMIBC

**Figure 1:**
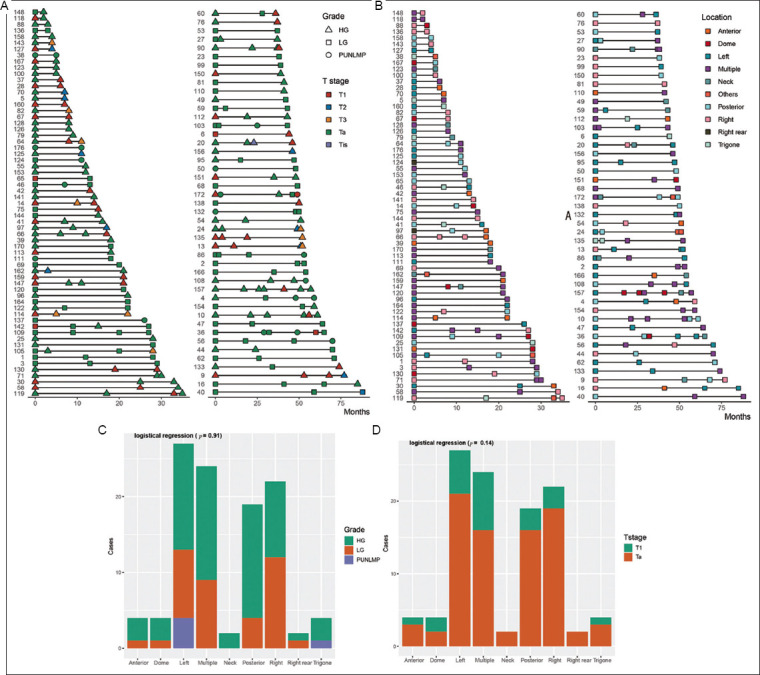
Patient characteristics. (A) Intravesical locations of patients’ primary and recurrent tumors. (B) Stages and grades of primary and recurrent tumors. (C&D) Tumor locations in terms of (C) grade and (D) stage. Abbreviations: HG: High-grade; LG: Low-grade; PUNLMP: Papillary Urothelial Neoplasm of Low Malignant Potential; Ta: Non-invasive papillary urothelial carcinoma; Tis: Tumor *in situ*.

**Figure 2:**
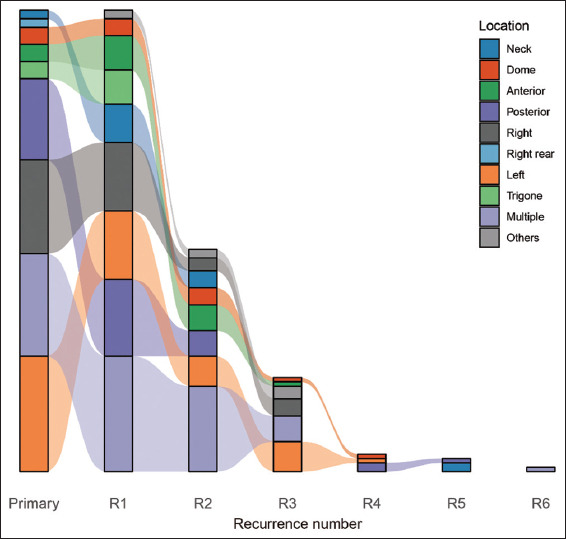
Changes in predilection locations with NMIBC recurrences. The most common sites of primary tumors are the left wall, multiple sites, and the right wall; with the first recurrence, they are multiple sites, the posterior wall, and the left wall; for the second recurrence, they are multiple sites, the left wall, and the posterior wall.

Among the 108 patients with NMIBC (83 men and 25 women), the median age was 70 years (range: 31–90 years), with a median follow-up duration of 28 months (range: 2–88 months). Figure 1 presents detailed information on tumor location, grade, and stage for primary tumors and each recurrence (Figure 1A and B), as well as tumor location stratified by stage and grade (Figure 1C and D). The results indicated that tumor location was not associated with tumor stage or grade. During the follow-up period, the average number of recurrences was three, with a maximum of six recurrences observed in a single patient. The most common sites of primary bladder cancer were the left lateral wall, multiple sites, and the right lateral wall (Figure 2). For the first recurrence, the top three most frequent locations were multiple sites, the posterior wall, and the left lateral wall; for the second recurrence, they were multiple sites, the left lateral wall, and the posterior wall (Figure 2).

### 3.2. Association between recurrence sites and original tumor sites

**Figure 3:**
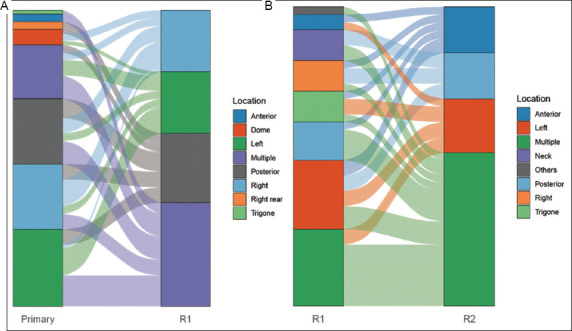
Tumor recurrence site tracing. (A) Comparison between the primary tumor sites and the first recurrence sites. (B) Comparison between the first recurrence sites and the second recurrence sites.

The origins of the first and second recurrence sites were traced, with a focus directed at the top three recurrence locations (Figure 3). Data showed that the common sites for primary tumors and first recurrences were the left lateral wall, right lateral wall, and posterior wall, the findings being consistent with typical bladder cancer locations. However, as the number of recurrences increased, the common tumor sites shifted. The main pattern persisted upon recurrence. Although the recurrence sites demonstrated a high concordance with those of the primary tumor, the incidence of multiple-site involvement increases. Overall, recurrence locations showed a high consistency with those of primary tumor sites.

### 3.3. Effect of recurrence numbers on recurrence interval and disease progression risk

**Figure 4:**
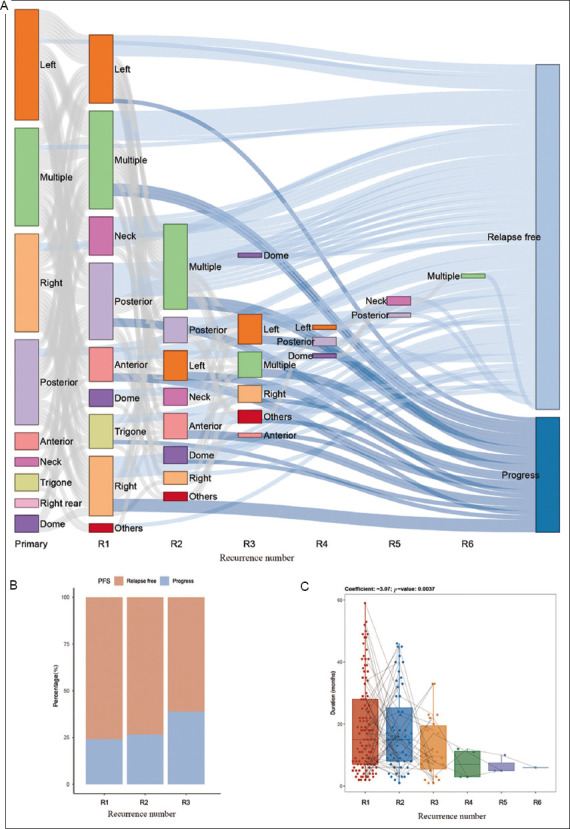
Characteristics of disease progression during tumor recurrence. (A) Tumor recurrence sites across recurrence numbers. (B) Proportion of progressive cases across recurrence numbers. (C) Tumor recurrence intervals. Abbreviation: PFS: Progression-free survival.

Tumor location changes were analyzed in this cohort of NMIBC patients. The results revealed no significant differences in pathological stage or grade among risk groups (Table 2), and most cases did not progress during recurrence (Figure 4A). However, as the number of recurrences increased, the proportion of progressive cases rose (Figure 4B), and the recurrence interval shortened (Figure 4C).

**Table 2:** Distribution of patients in each risk group

Parameters	Overall	Group 1 (neck+dome+rear+trigone)	Group 2 (left+right+posterior+anterior)	Group 3 (multiple)	*p*
*n*	108	12	72	24	
Age (year)	68.05 (11.75)	69.58 (13.03)	67.75 (11.27)	68.17 (12.96)	0.883
Gender					
Female	25 (23.1)	4 (33.3)	15 (20.8)	6 (25.0)	0.618
Male	83 (76.9)	8 (66.7)	57 (79.2)	18 (75.0)	
Grade					
HG	66 (61.1)	9 (75.0)	42 (58.3)	15 (62.5)	0.508
LG	37 (34.3)	2 (16.7)	26 (36.1)	9 (37.5)	
PUNLMP	5 (4.6)	1 (8.3)	4 (5.6)	0 (0.0)	
T stage					
T1	24 (22.2)	3 (25.0)	13 (18.1)	8 (33.3)	0.288
Ta	84 (77.8)	9 (75.0)	59 (81.9)	16 (66.7)	

Notes: Age data are expressed in mean (SD), while data of gender, grade, and T stage are given in number (percentage); continuous variables (*e.g*., age) were compared using one-way ANOVA, while categorical variables (*e.g*., gender, tumor grade, and T stage) were analyzed using the Chi-square test or Fisher’s exact test, depending on the expected cell counts. A p<0.05 was considered statistically significant, indicating potential clinical relevance.

Abbreviations: HG: High-grade; LG: Low-grade; PUNLMP: Papillary Urothelial Neoplasm of Low Malignant Potential, Ta: Non-invasive papillary urothelial carcinoma.

### 3.4. Impact of tumor location on recurrence interval and prognosis

**Figure 5:**
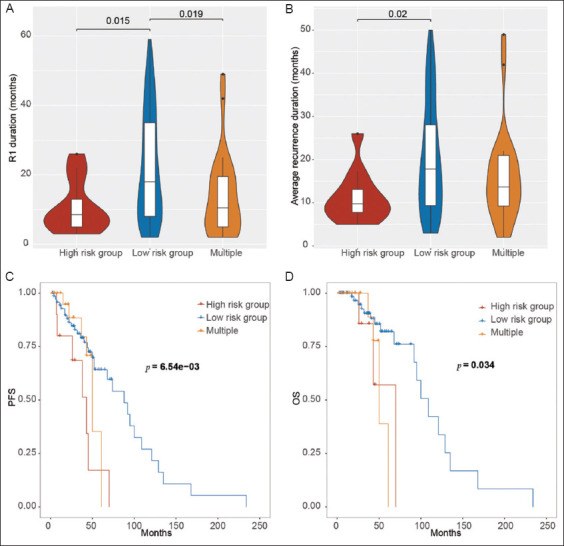
Impacts of recurrence on prognosis. (A) Recurrence duration for the first recurrence across three groups. (B) Average recurrence duration across three groups. (C) Progression-free survival (PFS) analysis. (D) Overall survival (OS) analysis. Notes: High risk group: Neck, dome, right rear wall, and trigone; Low risk group: Left, right, posterior, and anterior; Group 3: Multiple sites.

The precise location information for each primary and recurrent tumor was collected, and the impact of tumor location on prognosis was evaluated. Tumors located in the bladder neck, dome, right posterior wall, or trigone were associated with shorter recurrence-free intervals for both the first relapse (*p* = 0.015; Figure 5A) and the average recurrence interval (*p*=0.02; Figure 5B). In addition, tumors at multiple sites were linked to shorter intervals for the first relapse (*p*=0.019; Figure 5A). Tumors in these high-risk areas also demonstrated prognostic significance, being associated with poorer progression-free survival (*p*<0.001; Figure 5C) and overall survival (*p*=0.034; Figure 5D).

## 4. Discussion

Bladder cancer has specific predilection sites, with a prominent feature of NMIBC being its repeated recurrences. Nonetheless, few longitudinal studies examined the evolution of clinical features during the pathogenesis of NMIBC. The current study found that primary tumors predominantly affected the left lateral wall, multiple sites, and the right lateral wall. For the first recurrence, the most common sites were multiple sites, the posterior wall, and the left lateral wall; for the second recurrence, they involved multiple sites, the left lateral wall, and the posterior wall; for the third recurrence, the top sites were the left lateral wall, multiple sites, and the right lateral wall. Overall, while the sites of NMIBC recurrence shifted over time, the dominant recurrence sites still concentrated in the left lateral wall and right lateral wall, posterior wall, and multiple sites, indicating that the recurrence locations are highly consistent with the primary tumor sites. One potential explanation for this distribution pattern is the prolonged exposure of these mucosal areas to urinary carcinogens.

By tracing the origin of recurrent tumors, we found that, in both the first and second recurrences, tumors frequently recurred at the same sites as the original tumor. For example, the top three recurrence sites (exclusive of multiple sites) during the first recurrence—the posterior wall, left/right lateral wall—were primarily linked to the corresponding primary tumor location. This recurrence pattern lingered in the second recurrence.

In the cohort of 108 patients, 40.7% cases eventually developed muscle-invasive bladder cancer (MIBC). Previous studies have identified distinct molecular modes of progression, including a radical subtype shift preceding progression associated with p53 alterations.^1^ In line with this model, the present results showed that all instances of disease progression took place within the first three recurrences. Moreover, the progression rate increased with the number of recurrences, suggesting a cumulative effect of molecular changes over time. These findings support the hypothesis that bladder cancer progression results from the incremental accumulation of molecular changes through repeated recurrences. Furthermore, the interval between recurrences shortened as their number increased, further indicating an accelerating disease trajectory. Notably, previous studies have suggested that progressive MIBC—developing from NMIBC—may have a poorer prognosis than primary MIBC.^6^-^9^ Taken together, the current findings suggest that the number of bladder cancer recurrences is a significant risk factor for both recurrence and progression.

Research by Mulders *et al*.^2^ showed that tumors situated in the prostatic urethra, bladder neck, posterior wall, and trigone were significantly associated with shorter recurrence-free intervals. Similarly, Vukomanovic *et al*.^4^ reported that tumors in the bladder neck might have a higher risk of recurrence after intravesical immunotherapy, while tumors in the lateral and posterior bladder walls might be at a higher risk of recurrence when treated with transurethral tumor resection alone. In the current study, the bladder neck, dome, right posterior wall, and trigone were identified as high-risk locations significantly associated with shorter recurrence-free intervals. One potential explanation is protracted contact between urine-shed tumor cells and the bladder mucosa at these sites. However, the precise mechanisms and factors influencing tumor recurrence in these high-risk locations remain unclear and warrant further investigation.

Previous studies have suggested that tumors arising from the dome are associated with higher risks of higher-grade diseases.^10^ Similarly, some tumors are associated with increased odds of progressing to higher pathological stages (pT3–4).^5^ Bladder neck involvement in non-muscle-invasive tumors has also been reported to carry an increased risk of progression.^11^,^12^ With regard to survival outcomes, Martin *et al*.^13^ showed that dome involvement was associated with worse overall survival, while Svatek *et al*.^3^ reported comparable findings for tumors involving the trigone.

Studies have shown that cancers originating from different locations often exhibit distinct clinical characteristics and genetic profiles. Examples include skin cancers, such as squamous cell carcinoma and basal cell carcinoma;^14^ digestive system cancers, such as gastric cancer,^15^ colon cancer,^16^ and cholangiocarcinoma;^17^ and nervous system tumors, such as glioma.^18^ This phenomenon may be ascribed to oncogenic DNA alterations that fail to exert transformative effects in all cellular contexts.^14^,^19^ Studies have suggested that the anatomical location of the cell of origin imparts a unique transcriptional state, rendering it susceptible to specific oncogenic insults.^20^ In other words, anatomical location may be a major determinant of how cells respond to specific oncogenes, suggesting that distinct transcriptional programs might pre-exist in cells, pertaining to their anatomical origins.

This study was of a retrospective nature and involved a limited sample size. As the number of recurrences increased, the number of eligible cases gradually dropped, which may have affected the robustness of the conclusions. Therefore, the findings should be interpreted with caution and require validation in larger, prospective cohorts. In addition, the underlying mechanisms remain poorly understood and warrant further investigation.

## 5. Conclusion

A longitudinal analysis was conducted on 108 cases of NMIBC from a single center. The results showed that bladder cancer exhibited specific predilection sites, and these sites remained relatively stable throughout the recurrence process. Recurrence sites often were in line with the original tumor locations or previous recurrence sites. In addition, the proportion of progressive cases increased with successive recurrences, while the recurrence intervals became shorter. Patients with tumors located in high-risk areas suffered from shorter recurrence intervals and poorer prognoses.

## Data Availability

All data are available from the corresponding author upon request.
